# Towards the use of functional near-infrared spectroscopy as an assessment tool in disorders of consciousness

**DOI:** 10.1162/IMAG.a.1217

**Published:** 2026-04-30

**Authors:** Karnig Kazazian, Matthew Kolisnyk, Garima Gupta, Jack de Jeu, Androu Abdalmalak, Adrian M. Owen

**Affiliations:** Western Institute of Neuroscience, Western University, London, Canada; Department of Physiology and Pharmacology, Western University, London, Canada; Department of Psychology, Western University, London, Canada

**Keywords:** functional near-infrared spectroscopy, disorders of consciousness, covert awareness, consciousness, brain injury, cognition

## Abstract

Functional near-infrared spectroscopy (fNIRS) has emerged as a promising neuroimaging tool for assessing patients with disorders of consciousness (DoC). While functional magnetic resonance imaging (fMRI) and electroencephalography (EEG) have advanced the detection of covert brain function, their use is often constrained by accessibility, medical and physical contraindications, and practical limitations. fNIRS offers a portable, safe, and cost-effective alternative capable of measuring hemodynamic responses at the bedside. In this perspective, we discuss the clinical motivation for integrating fNIRS into DoC patient assessments, summarize recent advancements in the application of fNIRS for examining brain function, and outline the clinical and technical advantages. We highlight key future directions of fNIRS research, including large-scale validation, multimodal integration, and the development of fNIRS-based brain-computer interfaces. Finally, we address the ethical imperative to ensure equitable access to neurotechnologies capable of detecting covert brain function. With continued methodological refinement and standardization, fNIRS may significantly transform the diagnostic, prognostic, and communicative landscape of DoC care.

## 1 Introduction

Over the last 20 years, advanced neuroimaging techniques have fundamentally altered our understanding of residual brain function in patients with disorders of consciousness (DoC) (Kazazian, Monti, et al., 2025). In particular, functional magnetic resonance imaging (fMRI) and electroencephalography (EEG) have enabled the detection of preserved awareness and higher-order cognitive function in this clinical population (Bodien et al., 2024; Kazazian & Owen, 2025). These modalities have also improved prognostic accuracy; that is, aiding in the prediction of long-term neurological and functional outcomes (Egbebike et al., 2022; Kazazian et al., 2023; Norton et al., 2023; Pan et al., 2020). As a result, major clinical bodies have now endorsed the use of these methods as an assessment tool in select cases where standard behavioral assessments are inconclusive (Giacino et al., 2018; Kondziella et al., 2020).

However, both fMRI and EEG have inherent limitations, particularly where patients with DoC are concerned. fMRI requires transportation to specialized imaging facilities and is limited by several patient-specific and medical contraindications (Weijer et al., 2016). EEG, while portable, primarily captures electrical rather than hemodynamic signals, is limited in its spatial resolution. and is susceptible to substantial interference from electrical noise (Kazazian, Monti, et al., 2025). These drawbacks have motivated the quest to find alternative approaches to reliably assess residual brain function.

To this end, functional near-infrared spectroscopy (fNIRS) has recently emerged as a practical and promising alternative for assessing DoC patients (Abdalmalak et al., 2021; Kazazian, Abdalmalak, et al., 2024; Kazazian, de Jeu, et al., 2025). fNIRS is an optical neuroimaging technique that detects hemodynamic changes associated with neural activity. fNIRS has several unique advantages: it is portable, it can be deployed at the bedside or in home settings, it has a favourable safety profile, and it provides adequate spatial and temporal resolution for detecting task/stimulus-evoked or resting-state brain activity (Ferrari & Quaresima, 2012).

Here, we outline why fNIRS stands as an ideal tool for the assessment of DoC patients, explore its clinical and technical strengths and limitations, and outline future directions for research and its integration into routine care.

## 2 The Clinical Motivation of Advanced Neuroimaging

The term DoC is used to describe medical conditions that are characterized by disruptions in arousal and/or awareness following severe brain injury (Kazazian, Edlow, et al., 2024). These conditions span a continuum of responsiveness, ranging from coma (a state of unarousable unresponsiveness), to the vegetative state/unresponsive wakefulness syndrome (VS/UWS), where wakefulness is preserved but awareness is absent, to the minimally conscious state (MCS), where some aspects of awareness are evident (Edlow et al., 2020). Comprehensive descriptions of these conditions are provided elsewhere (Edlow et al., 2020; Kazazian, Edlow, et al., 2024). More recently, a novel category, termed covert awareness (Owen et al., 2006), or cognitive motor dissociation (Schiff, 2015), has been identified. In this condition, patients are able to demonstrate that their awareness is preserved through advanced neurotechnologies, while lacking any overt behavioral signs to support this (Bodien et al., 2024; Cruse et al., 2011; Owen et al., 2006). It is now recognized that up to 25% of behaviorally non-responsive DoC patients may retain covert awareness (Bodien et al., 2024; Kondziella et al., 2016; Monti et al., 2010).

DoC are also classified along a temporal continuum (Giacino et al., 2018). The acute phase of DoC refers to the first 28 days post-injury, a critical period when patients are often receiving life-sustaining interventions and decisions regarding the continuation or withdrawal of these measures are actively being considered. Patients who do not recover full responsiveness within this acute phase enter subacute to prolonged stages of DoC, often requiring ongoing assessment in rehabilitation hospitals, chronic nursing facilities, or at home (Edlow et al., 2020).

Behavioral examinations of awareness using standardized scales of responsiveness remain the cornerstone of DoC assessment (Bodien et al., 2022). While these tools are rigorous and capable of detecting even subtle signs of consciousness, they are inherently limited by several factors, including motor impairments, fluctuating arousal, sensory deficits, and examiner bias. As a result, they often fail to capture the full spectrum of responsiveness, leading to potential misdiagnoses (Schnakers et al., 2009). Accurate diagnosis of preserved consciousness is critical, as it informs goals of care decisions, shapes prognostic discussions with families and healthcare providers, and influences access to rehabilitative resources. The prognostic challenges in DoC care are equally pressing. Predicting whether a patient will regain consciousness, achieve functional independence, or remain in a prolonged state of unresponsiveness carries profound implications for medical decision-making (Fischer & Edlow, 2024). As it stands, no tools used in routine clinical practice can reliably predict recovery of consciousness, or long-term functional outcomes, in patients with acute or prolonged DoC.

Given these diagnostic and prognostic challenges, there is a clear need for objective, accessible tools for assessing brain function beyond behavioral observation.

## 3 Functional Near-infrared Spectroscopy: Principles and Mechanisms

fNIRS is an advanced neuroimaging tool that is often described as the optical equivalent of fMRI (Ayaz et al., 2022; Scholkmann et al., 2014). Near-infrared light penetrates biological tissue, including the scalp, skull, and cerebral cortex, and is differentially absorbed by oxygenated (HbO) and deoxygenated (HbR) hemoglobin. By using at least two wavelengths of light, fNIRS can estimate relative local changes in HbO and HbR concentrations (Scholkmann et al., 2014). Moreover, these hemodynamic changes reflect neurovascular coupling, wherein increased neuronal activity drives a localized surge in cerebral blood flow and oxygenation. Thus, fluctuations in HbO and HbR serve as an indirect proxy for underlying neural activity (Boas et al., 2014; Ferrari & Quaresima, 2012).

**Fig. 1.**
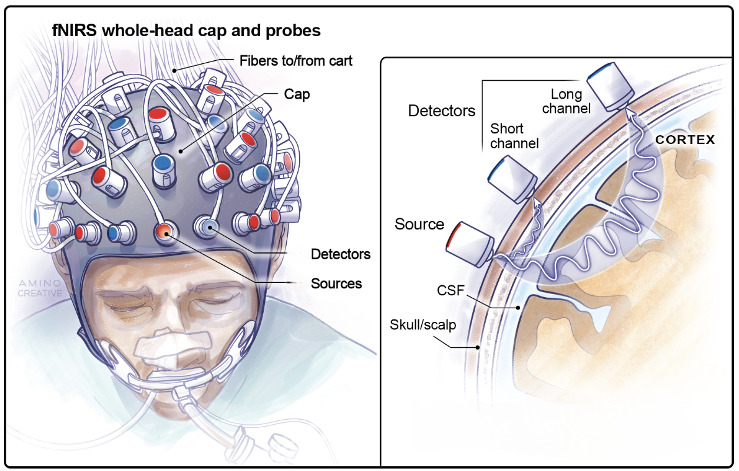
Schematic of an fNIRS whole-head cap with sources and detectors positioned to measure cortical hemodynamics. Near-infrared light penetrates the scalp and skull in a banana-shaped path, reaching the superficial cortex before being reflected back to one or more detectors. Light is differentially absorbed by oxygenated (HbO) and deoxygenated (HbR) hemoglobin, allowing estimation of regional changes in cerebral oxygenation. Adapted from Kazazian, Abdalmalak, et al. (2024) with permission.

An fNIRS montage consists of light sources that emit near-infrared light (i.e., laser diodes or LEDs) and detectors, which are secured on the scalp typically using a fitted cap (Fig. 1). As the light propagates through cortical tissue, it is scattered and absorbed following a probabilistic diffusion pattern (often described as ‘banana shaped’) en route to one or more detectors. The difference between the emitted and detected light intensity is then converted to optical density via the modified Beer-Lambert law and used to calculate relative changes in HbO and HbR over time (W. L. Chen et al., 2020). The depth of tissue measured depends on the source-detector separation, where so-called ‘long-channels’ (i.e., ~3 cm apart) are sensitive to cortical hemodynamics, while ‘short channels’ (i.e., <1 cm apart) primarily measure signals from extracerebral hemodynamics (Brigadoi & Cooper, 2015). While considerable variation exists in preprocessing protocols (Yücel, Luke, et al., 2024), common steps include: (1) removal of bad channels (i.e., source-detector pairs with a signal-to-noise ratio < 8); (2) motion correction (e.g., wavelet filtering or outlier-based methods); (3) bandpass filtering to remove slow drifts and physiological noise; and (4) short-channel regression to remove scalp hemodynamics (Huppert et al., 2009; Kazazian, Abdalmalak, et al., 2024).

## 4 fNIRS in the Assessment of DoC: State of the Science

**Fig. 2.**
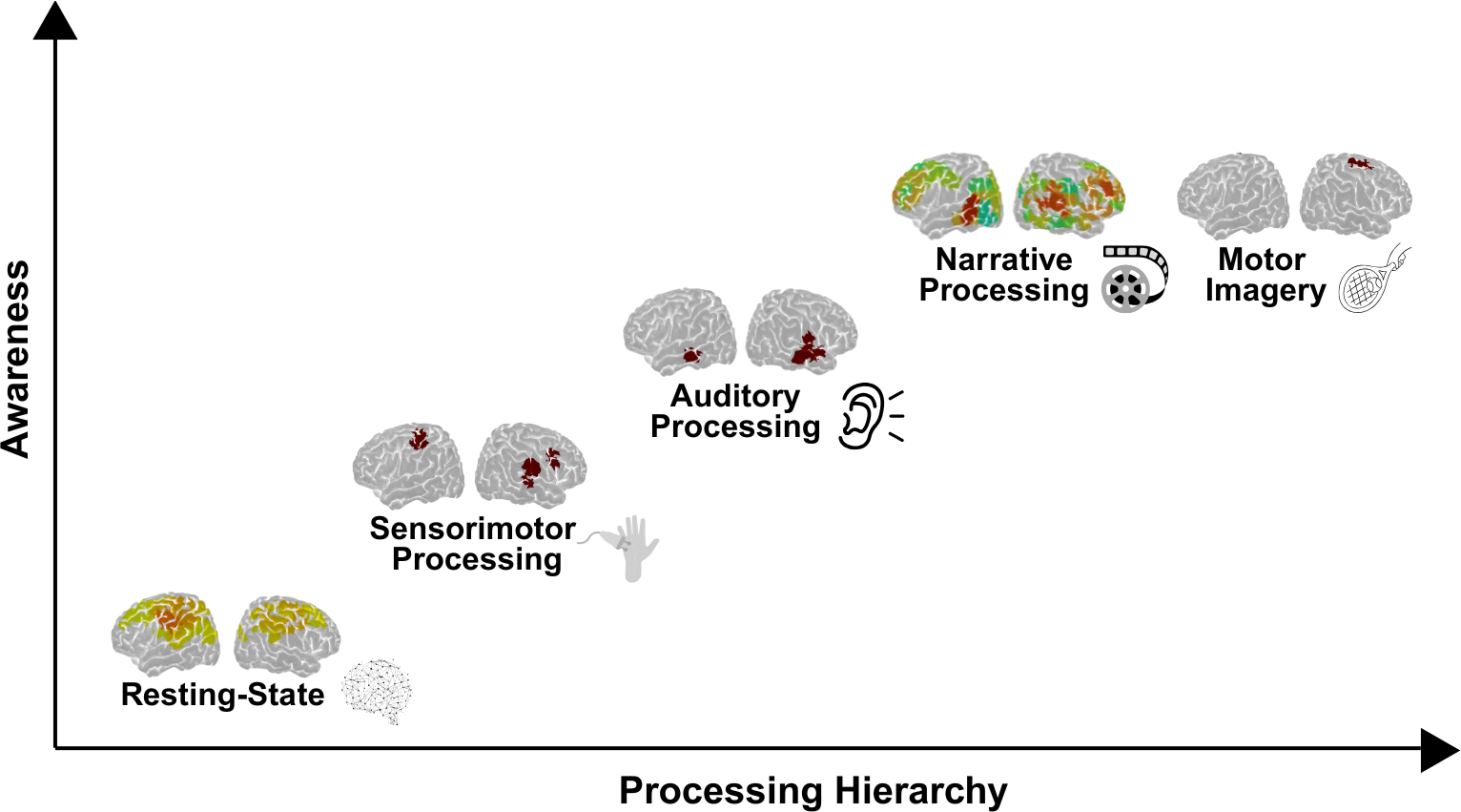
Hierarchical organization of neural processing in disorders of consciousness. The figure depicts a conceptual hierarchy of brain activity ranging from intrinsic resting-state networks to more complex, task-driven processes (sensorimotor, auditory, and narrative processing, culminating in motor imagery). The y-axis represents increasing levels of awareness, while the x-axis reflects the complexity of the processing hierarchy. Data shown are representative of general patterns across studies and do not reflect an individual patient. The figure is intended to illustrate the conceptual progression of brain activation rather than specific empirical results.

Broadly speaking, three general fNIRS approaches have been used to assess preserved brain function in DoC patients: (1) active mental imagery tasks to identify volitional command following, (2) passive sensory tasks to detect automatic neural responses, and (3), resting-state analyses to examine intrinsic brain connectivity. A summary of select studies is provided in Table 1. Representative results are outlined in Figure 2 for different neuroimaging paradigms in a single DoC patient.

**Table 1.** Select fNIRS publications with DoC patients.

Author (year)	Task category	Study cohort	Brief summary of main findings
Abdalmalak et al. (2020)	Command following	1 VS, 4 MCS, 1 LIS; prolonged	Three patients showed brain activity in response to commands, four with machine learning, and two with both methods—both of whom also demonstrated concordant activation on fMRI.
Kazazian et al. (2024)	Command following, passive, and rest	1 coma, 1 VS/UWS, and 1 MCS-; acute	An acutely brain-injured patient showed evidence for motor imagery and residual speech processing, while others showed significant response to only one of the tasks.
Kazazian et al. (2025)	Command following	12 coma, 15 VS/UWS, 5 MCS-; acute	8/32 patients (25%) were able to willfully modulate their brain activity when instructed to imagine playing a game of tennis.
Kempny et al. (2016)	Command following	10 HCs, 5 VS/UWS, and 11 MCS; prolonged	60% of HCs and 36% of patients showed an increase in HbO and a decrease in HbR during motor imagery. The opposite response was noted in 20% of HCs and 43% patients.
Li et al. (2021)	Command following + BCI	6 HCs, 5 MCS (2 MCS+, 3 MCS-); prolonged	Trained a classifier using HCs to decode “yes” (motor imagery) and “no” (rest) to five questions. When applied in patients, 3 of 5 patients showed task-evoked HbO increases and HbR decreases, with one patient providing consistent answers to all questions.
Si et al. (2023)	Command following + BCI	10 VS, 9 MCS; prolonged	MCS patients showed increased HbO in the pre-motor cortex during “yes” (motor imagery) and no activation during “no” (rest) responses. A VS/UWS patient showed consistent task-evoked HbO increases. HbO responses correlated with CRS-R scores at testing but not with recovery.
Wang et al. (2025)	Command following	30 VS, 20 MCS-, 20 MCS+; prolonged	Four VS/UWS and three MCS- patients showed significant evidence for motor imagery. At the 6-month follow-up, these seven patients were more likely to have a favorable outcome.
Zarifkar et al. (2025)	Command following	13 MCS, 23 VS; acute	In response to a motor imagery task, 62% of MCS patients and 35% of VS/UWS patients showed an increase in HbO and a decrease in HbR in frontal or premotor regions. VS/UWS patients with positive neural responses were numerically more likely to exhibit clinical signs of recovery within 1 week.
Zeng et al. (2025)	Command following	70 HC, 37 MCS, 26 VS; prolonged	In response to a motor imagery task, HCs showed robust HbO increases across motor, premotor, and occipital cortices. While MCS patients showed a weak response, VS/UWS patients showed none. The left premotor and bilateral motor cortices showed more activity in MCS than VS patients, and this activity predicted functional recovery at 6-months.
Bicciato et al. (2022)	Passive	6 HCs, 6 unresponsive, 9 partially responsive, 7 responsive; acute	Music-evoked low-frequency oscillations in the left prefrontal cortex were higher in HCs and partially responsive patients than unresponsive patients. These oscillations were significantly correlated with GCS and were significantly lower when comparing the poor outcome group to HCs.
Lu et al. (2023)	Passive, command following	15 HC, 14 VS, 4 MCS; prolonged	At the group level, HCs showed decreased HbO and HbR in the prefrontal cortex during both passive name hearing and imagined right-hand movement. Prolonged DoC patients showed the opposite pattern, with increased HbO and HbR.
Zhang et al. (2025)	Passive	7 UWS, 8 MCS; prolonged	During acute pressure stimulation, patients with DoC showed significantly increased functional connectivity between the primary somatosensory, motor, and dorsolateral prefrontal cortices.
He et al. (2024)	Rest	10 VS/UWS, 8 MCS; prolonged	VS patients showed higher connectivity strength in the left occipital lobe and longer characteristic path length, whereas MCS patients exhibited higher global efficiency. Combining these features with a KNN classifier discriminated between the patient groups with 89% accuracy.
Liu et al. (2023)	Rest	24 HCs, 12 MCS, 11 VS/UWS; prolonged	Prefrontal connectivity was highest in HCs and lowest in VS/UWS patients. Relative to HCs, short- and long-distance connections were reduced in VS/UWS patients, only long-distance connections were reduced in MCS. Compared to VS/UWS, short-distance connections were reduced in MCS. Connections within the right BA10, and between the left BA46 and right BA10 best distinguished MCS from VS/UWS.
Luo et al. (2024)	Rest	13 HCs, 10 DoC; prolonged	Functional connectivity, especially using HbO signals, was higher in HCs than patients. Logistic regression models based on network-specific connectivity features showed high classification accuracy for predicting arousal in patients, with AUCs of 93% for the sensorimotor network, 92% for the visual network, and 88% for visual-frontoparietal connectivity.

Studies are grouped by task category (command following, passive, and rest) and listed alphabetically by first author within each category.

AUC = Area Under the Curve, BCI = Brain-Computer Interface, CRS-R = Coma Recovery Scale–Revised, DoC = Disorders of Consciousness, fMRI = Functional Magnetic Resonance Imaging, GCS = Glasgow Coma Scale, HbO = Oxyhemoglobin, HbR = Deoxyhemoglobin, HCs = Healthy Controls, LIS = Locked-In Syndrome, MCS = Minimally Conscious State, UWS = Unresponsive Wakefulness Syndrome, VS = Vegetative State.

### 4.1 Command following tasks

In command-following paradigms^15^, patients are instructed to engage in a mental imagery task that requires intentional control of brain activity in response to external instructions. Given these requirements, positive neuroimaging results rely on the patient’s active participation, which is absent if they lack awareness (Fernández-Espejo & Owen, 2013). Pioneering fNIRS studies have demonstrated that covert awareness can be detected using such motor imagery tasks in some prolonged DoC patients (Abdalmalak et al., 2020; Kempny et al., 2016; Molteni et al., 2013). Subsequent studies have identified distinct response patterns in healthy controls, MCS, and VS/UWS patients, and related neuroimaging results to 6 month outcomes in the patient groups (Zeng et al., 2025). In the largest command following study to date, covert awareness was identified in 7 of 50 DoC patients who lacked behavioural evidence of awareness (Wang et al., 2025). Importantly, these 7 patients were also more likely to have a favorable outcome. Increasingly, fNIRS is being used at the patient bedside in acute care settings (Kazazian, Abdalmalak, et al., 2024; Zarifkar et al., 2025). Indeed, command-following tasks have recently been used to detect covert awareness in critically-ill brain-injured patients in the ICU, first in a pilot proof-of-concept study (Kazazian, Abdalmalak, et al., 2024) and later in larger group studies (Kazazian, de Jeu, et al., 2025; Zarifkar et al., 2025).

More recently, on a prospective study of 32 critically ill ICU patients with acute severe brain injury, fNIRS detected willful brain activity consistent with covert awareness in 25% of patients who showed no behavioral signs of command following. These findings demonstrate that bedside fNIRS can identify preserved consciousness in the acute setting, offering a critical safeguard against premature withdrawal of life-sustaining treatment (Kazazian, de Jeu, et al., 2025).

In addition to identifying covert awareness, mental imagery tasks are able to provide a basic form of communication for DoC patients. By assigning distinct imagery tasks to “yes” or “no” responses (for example, imagining playing tennis for “yes” and navigating one’s home for “no”), patients can alter their patterns of brain activity to convey answers to binary questions (Monti et al., 2010). fNIRS has already been used in this way to facilitate communication with locked-in patients (Abdalmalak, Milej, Norton, et al., 2017; Gallegos-Ayala et al., 2014), as well as covertly or minimally-aware individuals with prolonged DoC (Li et al., 2021; Si et al., 2023).

However, these paradigms face important limitations: command-following depends on intact language comprehension, sustained attention, and motivation, functions frequently impaired in severe brain injury. As such, a negative result cannot reliably indicate a lack of awareness (Fernández-Espejo & Owen, 2013; Kazazian & Owen, 2025). Furthermore, although a few studies have reported preliminary estimates of diagnostic performance (e.g., Wang et al. (2025), who reported 68.4% sensitivity [13/19 MCS+ patients] and 84.1% specificity [37/44 VS/UWS and MCS– patients]), sample sizes remain modest and clinical validation across different patient populations is ongoing. Consequently, larger prospective studies are required to establish robust and generalizable estimates of sensitivity and specificity across different clinical subgroups.

### 4.2 Passive paradigms

Passive paradigms assess brain activity in response to external stimuli that do not require active participation on the part of the patient. These paradigms provide information about preserved brain functioning, and by proxy, may serve as an index of the extent of injury (Norton et al., 2023; Sokoliuk et al., 2021). Previous fNIRS studies have used various paradigms to assess passive neural responses in DoC, using tactile (Zhang et al., 2025), sensorimotor (Kazazian, Abdalmalak, et al., 2024), narrative (Kolisnyk et al., 2024; Laforge et al., 2025), and auditory stimuli (Bicciato et al., 2022; Lu et al., 2023; H. Yang et al., 2025). These paradigms have shown clear differences in neural responses between VS and MCS patients (Kurz et al., 2018; Lu et al., 2023; H. Yang et al., 2025). Passive paradigms have also been used to assess low-level speech processing and higher-order language comprehension in acutely ill brain-injured patients in the ICU (Kazazian, Abdalmalak, et al., 2024). However, the prognostic relevance of such responses remains to be fully elucidated.

Passive paradigms are less cognitively demanding and therefore more inclusive, but interpretation remains challenging: preserved sensory or language processing does not necessarily infer awareness (Fernández-Espejo & Owen, 2013; Kazazian & Owen, 2025). Further work is needed to determine which passive responses predict future recovery and how they should inform clinical decision-making.

### 4.3 Resting state

Resting-state paradigms are particularly valuable because they reveal intrinsic patterns of brain connectivity in the absence of external stimuli. In some instances, these patterns may reflect preserved consciousness and a capacity for neurological recovery (Kazazian, Monti, et al., 2025). A considerable number of fNIRS studies have sought to characterize DoC patients based on resting-state data (H. Chen et al., 2023; He et al., 2024; Liu et al., 2023; Luo et al., 2024), and differentiate between states of consciousness (e.g., VS vs MCS) using graph-based metrics (Luo et al., 2024) and network-based approaches (Liu et al., 2023). Furthermore, resting-state fNIRS has shown some potential for tracking treatment effects. For example, increases in global and regional connectivity have been reported, especially in frontal-parietal circuits, that align with improvements on behavioral assessments following interventions like deep brain stimulation (Y. Yang et al., 2023) and vagus nerve stimulation (Gao et al., 2025).

Yet, resting-state metrics remain difficult to standardize, with considerable variability in acquisition, preprocessing, and analytical methods across studies (Kazazian, Edlow, et al., 2024). Moreover, the relationship between network connectivity and subjective awareness is indirect, highlighting the need for multimodal correlation and longitudinal validation.

## 6 Clinical and Technical Considerations

### 6.1 Clinical advantages of fNIRS

fNIRS can be deployed at a patient’s bedside within hospitals and in community settings. Typically, setup begins by selecting the appropriate cap size based on the patient’s head circumference and pre-populating it with sources and detectors, which typically takes 10–20 minutes. An additional 10–20 minutes are required for cap placement, adjustment, and system calibration at the bedside. The same setup is compatible with mechanically ventilated patients and can be adapted for those who are sitting upright or lying flat.

With its logistical simplicity and portability, fNIRS eliminates the risks associated with transporting medically complex patients to in-hospital fMRI suites, making it particularly advantageous in acute care (Kazazian, Monti, et al., 2025; Weijer et al., 2016). Importantly, fNIRS can also be deployed in the homes or care homes of patients with prolonged DoC. This approach removes the need for complex logistical arrangements that are often required to transport community-dwelling patients to MRI facilities. This portability and ease of use allows fNIRS assessments to be conducted in rural and remote settings that would otherwise have no access to advanced imaging.

Furthermore, fNIRS systems are quiet, making it not only more tolerable for patients than the loud scanning environments of fMRI but also better suited for auditory, language-based, and naturalistic paradigms where scanner noise can interfere with stimulus processing (Bandettini et al., 1998; Plichta et al., 2011; Santosa et al., 2014). fNIRS is also entirely non-invasive (i.e., does not require radioactive tracers) and is suitable for patients with typical MRI contraindications, including metallic implants (e.g., shunts, pacemakers, cochlear implants). Finally, the ease of set-up and affordability of fNIRS allows for serial testing and continuous monitoring, which are both essential for reliably capturing fluctuations in arousal and awareness (Kazazian, Edlow, et al., 2024).

### 6.2 Technical advantages of fNIRS

fNIRS has slightly poorer spatial resolution than fMRI and a lower temporal resolution than EEG (Pinti et al., 2020). Nevertheless, fNIRS montage can reliably detect activity within, and differentiate activity between, cortical regions, including the supplementary motor area and the premotor cortices, which are commonly targeted in mental imagery paradigms (Abdalmalak, Milej, Diop, et al., 2017). fNIRS provides a higher sampling rate (up to 10 Hz) than fMRI (1–3 Hz) (Pinti et al., 2020), and can capture the hemodynamic response with fine granularity, enabling the assessment of onset, peak and duration following stimulus presentation. Furthermore, fNIRS is less susceptible to small movements (e.g., blinking, jaw clenching and swallowing) than either fMRI or EEG, making it well-suited to clinical contexts (Abdalmalak et al., 2021).

### 6.3 Clinical limitations of fNIRS

To date, fNIRS has remained a research tool rather than a clinical measure, largely because established clinical protocols are lacking, varying methods of data acquisition are employed, and significant variability exists in signal preprocessing and analysis pipelines. As with all neuroimaging modalities, the absence of standardized analysis procedures increases the likelihood of false positives and negatives being reported with fNIRS (Kazazian, Edlow, et al., 2024). For example, in the absence of consistent preprocessing and statistical thresholds, artifacts or random fluctuations in fNIRS signal can be misinterpreted as positive responses. Moreover, data acquisition can be challenging in patients with physical barriers to optode placement, including craniocerebral or c-spine injuries, external ventricular drains or craniectomies (Kazazian, Abdalmalak, et al., 2024). fNIRS may also be unsuitable in patients with subdural hematoma, which can affect the diffusion and absorption of near-infrared light. Time, space, and movement-related restrictions may place additional constraints in an acute care setting. Signal quality can also vary based on patient-specific characteristics, such as hair type and skin tone (Patel et al., 2024), although recent advances in software and hardware have substantially reduced the effect of these factors (Yücel, Anderson, et al., 2024).

### 6.4 Technical limitations of fNIRS

fNIRS does not provide direct anatomical information and therefore cannot localize the origin of hemodynamic responses with absolute certainty (Quaresima & Ferrari, 2016). Although tools such as AtlasViewer allow for spatial registration of fNIRS montages onto standardized brain templates, the precise identification of specific regions still requires co-registration with an anatomical MRI, especially in patients with structural brain changes (Desikan et al., 2006; Quaresima & Ferrari, 2016; Tzourio-Mazoyer et al., 2002). In addition, due to an average depth sensitivity of ~1.5 cm, fNIRS is restricted to detecting activity within superficial cortex and cannot reach midbrain regions (Quaresima & Ferrari, 2019). Furthermore, fNIRS signals are susceptible to contamination from systemic physiology, including fluctuations in heart rate and respiration (Abdalmalak et al., 2022). Short-channel regression is often used to mitigate these effects, but cannot fully eliminate the risk of physiological interference, making it difficult to separate stimulus-evoked hemodynamic responses from physiological noise (Abdalmalak et al., 2022). To address this, systemic physiology is often recorded during the fNIRS assessment using non-invasive and clinically standardized devices, such as capnographs and blood pressure monitors.^56^ Although fNIRS provides (slightly) superior temporal resolution than fMRI, it lacks the sensitivity required to capture rapid neural dynamics. Consequently, fNIRS-based paradigms require task blocks (10–30 seconds) to detect a robust response (Abdalmalak et al., 2021).

## 7 Future Directions

**Fig. 3.**
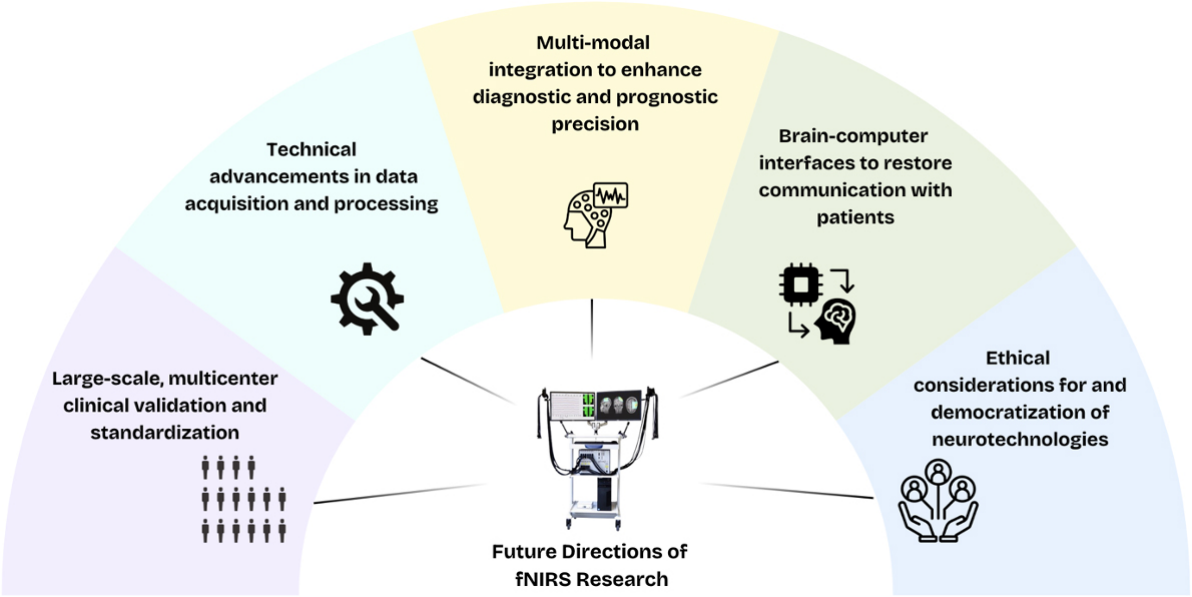
Future directions of fNIRS research. Schematic overview highlighting key priority areas for advancing the clinical and scientific utility of fNIRS, including large-scale clinical validation, technical advancements, multi-modal integration, brain–computer interfaces, and ethical considerations. Image provided by NIRx.

Looking ahead, research efforts in five key areas are essential to advance the clinical utility of fNIRS in DoC patients (see Fig. 3).

### 7.1 Large-scale clinical validation

First, there is a need for large-scale, multicenter validation studies. Most fNIRS work to date in DoC patients is limited by small sample sizes, single-center designs, and varied methodologies and systems, hindering reproducibility and clinical adoption. Future work should prioritize standardized paradigms, harmonization of preprocessing pipelines, and common behavioural and outcome measures. These protocols must span acute, subacute, and chronic time points to fully capture the evolving trajectory of fluctuating brain function and recovery in DoC. A critical next step is to prospectively evaluate the prognostic value of fNIRS findings, such as the command-following and passive-response signatures shown to differentiate outcomes within routine clinical decision-making workflows. Demonstrating that fNIRS-positive patients consistently recover better would strongly support incorporation into prognostic guidelines. To date, no clinical guidelines endorse the use of fNIRS as an assessment tool in DoC, likely due to the lack of large-scale research and the predominance of underpowered studies. Clinical uptake remains limited due to lack of personnel, cost barriers, and the relatively immature nature of the technology (Kazazian, Monti, et al., 2025).

### 7.2 Technical innovations

In parallel with clinical validation, technical innovation is needed to optimize signal sensitivity and reduce noise. Advances in time-resolved fNIRS (Abdalmalak, Milej, Diop, et al., 2017), high-density optode arrays (Anderson et al., 2025), and cap systems targeting specific brain regions are key areas that would increase the use of fNIRS. DoC-specific challenges such as skull defects after hemicraniectomy, mechanical ventilation, and variable tissue optical properties associated with cerebral edema require adaptive cap designs, robust real-time motion correction, and automated quality-assurance systems that can function in the ICU. Additionally, concurrent physiological monitoring (e.g., end-tidal CO_2_, blood pressure) and systemic physiology regression should be more fully integrated into studies in order to mitigate contamination from extracerebral signals (Abdalmalak et al., 2022). Real-time signal quality feedback and automation of artifact detection should also be used to increase reliability, especially in clinical settings (Pollonini et al., 2016).

### 7.3 Multimodal integration

Multimodal integration is likely to play a key role in advancing fNIRS toward more complete clinical integration. In particular, pairing fNIRS with EEG can enable simultaneous or consecutive tracking of electrical and hemodynamic responses, offering a more comprehensive picture of brain function than either modality alone (Laforge et al., 2025; Othman et al., 2021; Su et al., 2023). For example, in cases where mental-imagery EEG responses are ambiguous or absent, a positive fNIRS signal may provide convergent evidence of covert awareness—particularly relevant in acute DoC patients where positive results may have significant clinical implications.

Multimodal integration can also increase diagnostic confidence at the individual patient level, especially when findings from one technique are ambiguous. Pupillometry is another emerging complimentary assessment; during tasks such as mental arithmetic, measurement of pupil dilation alongside hemodynamic responses with fNIRS may provide insight into coordinated cognitive effort (Yeung et al., 2021). Although multimodal acquisitions can be technically demanding, integrated platforms and synchronized systems are becoming increasingly available.

Importantly, the established prognostic utility of fMRI and EEG also provides a natural framework for the validation of fNIRS-based markers. Rather than positioning fNIRS as an independent prognostic modality at this stage, fNIRS signals can be benchmarked against well-characterized fMRI network measures and EEG-derived indices of consciousness and outcome. Such cross-modal validation enables assessment of whether fNIRS captures convergent neural processes already known to carry prognostic significance, while leveraging its practical advantages in bedside and acute care settings. This multimodal grounding is likely to be a critical step in translating fNIRS from a promising research tool into a clinically interpretable adjunct within established prognostic workflows.

### 7.4 Brain-computer interfaces

Brain computer interfaces (BCIs) are one of the most transformative, yet underexplored, clinical applications of fNIRS. While proof-of-concept studies have demonstrated that some behaviourally non-responsive patients can answer “yes” or “no” questions using mental imagery paradigms, these studies remain largely experimental (Abdalmalak, Milej, Norton, et al., 2017; Li et al., 2021; Si et al., 2023). EEG-based BCIs, for instance, have proven highly successful in healthy participants, but their translation into the DoC population has been limited by several factors, including susceptibility to artefacts from muscle activity and eye movements, reduced signal quality in the presence of structural brain injury, and difficulties in distinguishing true volitional responses from spontaneous fluctuations in neural activity (Naci et al., 2012).

fNIRS-BCIs may be especially advantageous for patients with extensive cortical injury or motor impairment, where hemodynamic responses remain preserved even when electrophysiological signals are degraded. Development of hybrid fNIRS-EEG BCIs also offers the possibility of detecting intentional responses even when only one modality produces a usable signal. Developing reliable fNIRS-based BCIs requires that advances in signal processing be integrated with adaptive task designs, and machine-learning approaches to enhance sensitivity and robustness at the single-patient level. However, effective clinical deployment also hinges on careful patient selection, as factors such as fluctuating arousal, sedation, or severe delirium may compromise a patient’s ability to engage with the BCI. That said, the portability of fNIRS, its ability to generate rapid results, and its capacity for detecting real-time neural responses, position it as the most suitable neuroimaging modality for BCI applications in patients with DoC.

Although fNIRS-based BCIs often rely on block-design paradigms to achieve robust signal detection, this does not preclude their use for communication or neurofeedback. Similar to real-time fMRI, emerging real-time GLM-based fNIRS approaches enable online detection of intentional neural responses, with communication protocols that prioritize accuracy and reliability over speed, which is an essential consideration in patients with disorders of consciousness. In this context, slower but dependable fNIRS-BCI systems may be particularly well suited for clinical deployment, where minimizing false positives is more critical than maximizing communication rate (Abdalmalak et al., 2021; Abdalmalak, Milej, Diop, et al., 2017; Abdalmalak, Milej, Norton, et al., 2017).

### 7.5 Ethical considerations

Finally, the use of fNIRS to detect preserved cognition and covert awareness in DoC raises complex ethical questions that extend beyond technical validation. For example, this technology remains limited to a small number of specialized centers worldwide (Helbok et al., 2022) and there is an imperative to democratize access through initiatives such as shared protocols and open-source analysis pipelines. Without such efforts, there is a risk that fNIRS will remain siloed in academic centers, accessible only to a privileged few. Furthermore, the analytical interpretation of fNIRS findings demands caution. A false-positive result risks offering false hope, while a false negative may lead to withholding life-saving therapies. With the latter in mind, the absence of an fNIRS response should never be considered an absence of brain function. Continued interdisciplinary collaboration between clinicians, scientists, and ethicists is needed for the adoption and refinement of rigorous standards in data acquisition, analysis, and communication of fNIRS results (Young et al., 2024).

Skin pigmentation represents an important and increasingly recognized source of bias in optical neuroimaging. Recent work has demonstrated reduced signal-to-noise ratios in individuals with darker skin tones, attributable to increased absorption of near-infrared light by melanin, highlighting a limitation of continuous-wave fNIRS systems that parallels longstanding issues in pulse oximetry (Yücel et al., 2025). However, these findings also indicate that pigmentation-related effects are not inherent to all fNIRS approaches. When high-sensitivity detectors were used, channel exclusion rates were substantially reduced across a large cohort, suggesting that advances in instrumentation can meaningfully improve data quality across skin tones (Yücel et al., 2025). Moreover, frequency-domain and time-domain fNIRS offer additional advantages by enabling improved separation of superficial and cortical signals and more accurate modeling of photon propagation, and prior work has shown minimal skin-tone–related effects on measurement accuracy with time-domain systems (Lacerenza et al., 2025).

## 8 Conclusion

fNIRS offers many clinically important advantages for detecting brain function in patients with DoC, particularly in settings where fMRI or EEG are impractical, unsafe, or inconclusive. Widespread adoption of fNIRS will depend on the development of robust training programs, shared data collection and analysis protocols, and ethical frameworks for the interpretation and disclosure of findings. Taken together, fNIRS has the potential to expand the current neurodiagnostic toolkit for patients with DoC, enabling clinicians to build a more comprehensive and precise understanding of residual brain function in this complex medical population.

## Data Availability

No data or code was used to generate this review paper.
